# Resilience and pain catastrophizing among patients with total knee arthroplasty: a cohort study to examine psychological constructs as predictors of post-operative outcomes

**DOI:** 10.1186/s12955-021-01772-2

**Published:** 2021-05-01

**Authors:** Vesta C. Nwankwo, William A. Jiranek, Cynthia L. Green, Kelli D. Allen, Steven Z. George, Janet Prvu Bettger

**Affiliations:** 1 Duke University School of Medicine, Durham, NC USA; 2 Department of Orthopaedic Surgery, Duke University School of Medicine, Durham, NC USA; 3 Department of Biostatistics and Bioinformatics, Duke University School of Medicine, Durham, NC USA; 4 Center to Accelerate Discovery and Practice Transformation, Durham VA Health Care System, Durham, NC USA; 5 Department of Medicine, Duke University School of Medicine, Durham, NC USA; 6 Department of Medicine and Thurston Arthritis Research Center, University of North Carolina, Chapel Hill, NC USA; 7 Duke Clinical Research Institute, Durham, NC USA

**Keywords:** Arthroplasty knee resilience pain catastrophizing function physical mental health outcomes

## Abstract

**Background:**

Patients’ psychological health may influence recovery and functional outcomes after total knee arthroplasty (TKA). Pain catastrophizing, known to be associated with poor function following TKA, encompasses rumination, magnification, and helplessness that patients feel toward their pain. Resilience, however, is an individual's ability to adapt to adversity and may be an important psychological construct that supersedes the relationship between pain catastrophizing and recovery. In this study we sought to identify whether pre-operative resilience is predictive of 3-month postoperative outcomes after adjusting for pain catastrophizing and other covariates.

**Methods:**

Patients undergoing TKA between January 2019 and November 2019 were included in this longitudinal cohort study. Demographics and questionnaires [Brief Resilience Scale (BRS), Pain Catastrophizing Scale (PCS), Knee injury and Osteoarthritis Outcome Score, Junior (KOOS, JR.) and Patient-Reported Outcomes Measurement Information System Physical and Mental Health (PROMIS PH and MH, respectively)] were collected preoperatively and 3 months postoperatively. Multivariable regression was used to test associations of preoperative BRS with postoperative outcomes, adjusting for PCS and other patient-level sociodemographic and clinical characteristics.

**Results:**

The study cohort included 117 patients with a median age of 67.0 years (Q1–Q3: 59.0–72.0). Fifty-three percent of patients were women and 70.1% were white. Unadjusted analyses identified an association between resilience and post-operative outcomes and the relationship persisted for physical function after adjusting for PCS and other covariates; in multivariable linear regression analyses, higher baseline resilience was positively associated with better postoperative knee function (β = 0.24, *p* = 0.019) and better general physical health (β = 0.24, *p* = 0.013) but not general mental health (β = 0.04, *p* = 0.738).

**Conclusions:**

Our prospective cohort study suggests that resilience predicts postoperative knee function and general physical health in patients undergoing TKA. Exploring interventions that address preoperative mental health and resilience more specifically may improve self-reported physical function outcomes of patients undergoing TKA.

**Supplementary Information:**

The online version contains supplementary material available at 10.1186/s12955-021-01772-2.

## Background

Total knee arthroplasty (TKA) is a procedure that is currently performed over 600,000 times annually in the United States [1]. Common indications for TKA include pain, disability, impact on daily function, and arthritic deformity of the knee such as osteoarthritis, rheumatoid arthritis, and other forms of arthritic deformity [2]. Patients with osteoarthritis account for approximately 95% of TKA cases each year [2, 3]. Typically, before surgery is considered, physicians will initiate a trial period of conservative therapies [4]. These can vary based on the type of arthritic insult, but can include weight loss, aerobic and anaerobic exercise, nonsteroidal anti-inflammatory drugs, and a variety of other treatments. Consideration of psychological interventions is not common and are not standard practice even preoperatively despite evidence to suggest their potential benefit.

Despite pain reduction and mobility improvements from TKA, research has indicated that patients undergoing TKA with higher levels of preoperative pain catastrophizing experience lower levels of function following surgery [5]. Similar to depression and anxiety, pain catastrophizing is a negative psychological construct that has received attention in orthopedics and other fields. Pain catastrophizing captures patients’ pain-related thoughts of rumination, magnification, and feelings of helplessness [5]. In chronic pain studies, pain catastrophizing has been cited as a vulnerability factor in the pathway to physical functioning whereas resilience mechanisms are thought to represent coping responses [6]. Additionally, a recent systematic review conducted in patients with TKA demonstrated a relationship between pain catastrophizing and increased chronic pain [7]. Higher levels of pain catastrophizing have also been linked to poor function [8], more postoperative pain [9], and more nighttime pain [9] consistently in other studies.

Unlike pain catastrophizing, resilience is a positive psychological construct that has recently gained more attention in orthopedics. This construct encompasses positive environmental and emotional characteristics that allow a person to endure adversity [10]. Optimism, independence, and protective family and community networks are also used to define this construct [11, 12]. Psychological resilience is inversely correlated with depression and facilitates adaptation to distressing events, such as psychological and physical trauma [13–15]. Patients who have suffered traumatic physical injuries (brain, spinal cord, and musculoskeletal) and engage in resilience-building programs return to work in a shorter amount of time and have improved self-efficacy [16]. Further, higher resilience may predict reductions in pain catastrophizing in chronic pain patients over time [17]. Only a few studies in the arthroplasty literature have attempted to define the association between resilience and post-surgical outcomes, specifically focusing on knee function and quality of life [18–20] with conflicting findings to date. Research on the association of resilience and outcomes specific to TKA is sparse. Further, it is unclear if stronger positive resilience would be associated with better post-surgical outcomes after accounting for negative psychological constructs such as pain catastrophizing. Clearly delineating these relationships could play an important role in how clinicians optimize patients prior to TKA.

To more clearly define the relationship between resilience and postoperative outcomes, we conducted a prospective cohort study to investigate whether preoperative resilience is predictive of postoperative knee function, general physical health, and general mental health 3 months after TKA, and whether any association identified would persist after adjusting for pain catastrophizing and patient sociodemographic and clinical characteristics. These two psychological constructs are often studied independent of one another and to our knowledge, this is the first study of patients with TKA to examine them together.

## Methods

### Study design and setting

In this prospective cohort study, patients were recruited from an outpatient orthopedic clinic of a large academic medical center from January 2019 to November 2019. Approval from the Institutional Review Board (IRB) was obtained prior to initiation of this study. Patient information was collected and stored within REDCap, a secure, web-based application platform [21].

### Patients and enrollment

Prior to a clinic appointment, the patient's electronic medical record was pre-screened for study eligibility. Following consent for surgery by one of four joint replacement surgeons in the outpatient orthopedic clinic, patients were informed of the opportunity to participate in a study of outcomes for patients undergoing TKA. If interested and study eligible, the study was explained, and the patient consented prior to leaving the clinic. Patients were eligible if they were able to read and write in English, able to provide written informed consent, 35–85 years old, and approved to undergo unilateral TKA by an orthopedic surgeon. We chose not to include patients younger than 35 years in order to select against patients with knee pathology related to congenital, traumatic, and developmental origins [22]. The maximum age of 85 years was chosen to minimize loss to follow-up based on the clinic’s experience with electronic data collection where patients were required to participate from home after surgery. Patients were excluded based on the following criteria: medically unstable presentation at time of consent (indicating a picture of shock or sepsis), TKA scheduled because of a fracture, malignancy or an infection, bilateral TKA, cognitive and/or neurological disorders that could interfere strongly with questionnaires and surveys. Patients were further excluded if their baseline measures were incomplete.

### Perioperative care

Total knee arthroplasty management criteria at the study’s institution are fairly standardized. On the day of surgery, patients were treated preoperatively with regional anesthesia to include a spinal block in addition to one or two additional peripheral nerve blocks. Postoperatively, in addition to the regional anesthesia blocks, patients received pain medications (e.g., Tylenol, nonsteroidal anti-inflammatories, and opioids) for breakthrough pain. Patients were discharged on a combination of these medications. For postoperative therapy, patients were weight-bearing as tolerated and began therapy on postoperative day zero. Aggressive range of motion was discouraged until at least 2-weeks post-surgery, allowing swelling to dissipate and the wound to heal.

### Data, sources, and procedures for collection

#### Data collection procedures

Baseline assessments were collected at least 7 days prior to the patient’s surgery date. Following study enrollment, patients completed a demographics survey capturing age, sex, race, ethnicity, marital status, employment status, years of education, and insurance type. The patient’s current overall pain intensity was assessed using a Pain Numeric Rating Scale (NRS). The Pain NRS is a single item response value on a scale of 0–10 with higher scores indicating increased intensity [23].

At baseline, patients were asked to complete four questionnaires. These questionnaires were repeated at 3-months following surgery. At the postoperative visit, a study investigator would systematically attempt to meet patients in person at their scheduled clinic visit with the operating provider (if scheduled). If no appointment was scheduled, study investigators would utilize email, then a phone call, which was followed by a mailed survey packet if no contact was made. All follow-up data were obtained no more than 1 week before or after each scheduled 3-month follow-up time point. When evaluating patient-reported function and pain, studies have shown that clinical services and the majority of change occurs within the 3-month postoperative period [24, 26].

#### Data and sources

Comorbidities have been shown to influence functional outcomes of following TKA. To account for these factors, baseline clinical information was collected via retrospective chart review of the electronic medical record. These data included a documented history or clinical diagnosis of depression, anxiety, and back pain (with specific category for low back pain). We also evaluated for the presence of diabetes by identifying patients with a history or clinical diagnosis of diabetes, or evidence of any of the following: blood sugar > 7 mmol/L or > 126 mg/dL on two or more fasting plasma glucose tests; blood sugar > 200 mg/dL on two or more oral glucose tolerance tests; blood sugar > 200 mg/dL on random plasma glucose test in the presence of increased urination, increased thirst, or unexplained weight loss; hemoglobin A1c ≥ 6.5%; chronic treatment with anti-diabetic medications, including insulin. Classification for a history or clinical diagnosis of diabetes for this study did not include gestational diabetes, glycemic disorders (e.g., hypoglycemia), or pre-diabetes.

Patients were evaluated for hypertension by determining if they had a history or clinical diagnosis of high blood pressure; or evidence of any of the following: hypertension, whether treated or untreated; blood pressure > 140 mm Hg systolic and/or > 90 mm Hg diastolic for patients without diabetes or chronic kidney disease; blood pressure > 130 mm Hg systolic and/or 80 mm Hg diastolic on at least two occasions for patients with diabetes or chronic kidney disease; currently prescribed medication for treatment of hypertension (e.g., Angiotensin-converting enzyme inhibitor, angiotensin receptor blocker, beta blocker and diuretic). Finally, patients were considered to have a history of cardiovascular disease if there was documentation of a history or clinical diagnosis of coronary artery disease, myocardial infarction, stroke, arrhythmia, valvular disease, or heart failure [24]. Body mass index (BMI), the American Society of Anesthesiologists (ASA) classification score (1 = a normal healthy patient, 2 = a patient with mild systemic disease, 3 = a patient with severe systemic disease, 4 = a patient with severe systemic disease that is a constant threat to life, 5 = a moribund patient who is not expected to survive without the operation and 6 = a declared brain-dead patient whose organs are being removed for donor purposes) [25], surgery type (primary vs. revision), previous TKA on the contralateral side, diagnostic criteria (knee arthritis etiology), smoking status, and pack years were also collected in the retrospective chart review.

Pain catastrophizing scores were calculated using the *Pain Catastrophizing Scale* (PCS) [5, 26]. This instrument incorporates common thoughts and reactions seen in pain catastrophizers: rumination (“I can’t stop thinking about how much it hurts”), magnification (“I worry that something serious may happen”), and helplessness (“There is nothing I can do to reduce the intensity of the pain”). This scale is useful because it analyzes recent pain-related thoughts. The PCS score is obtained by summing the values for all 13 items within the measure. Scores range from 0 to 52 and higher scores indicate increased pain catastrophizing.

Patient resilience scores were calculated using the *Brief Resilience Scale* (BRS) [11]. This 6-item scale was designed to succinctly assess a patient’s perception of their ability to “bounce back” in the setting of negative life events. This scale represents the concept of resilience most directly, whereas other scales are more reflective of the personality traits and strategies that patients utilize in order to increase their resilience. Items on the BRS are scored on a 5-point Likert Scale. The total BRS score (range 1–5) is an average of all of the items (after reverse coding 3 items) with higher scores indicating more resilient individuals.

The following standardized instruments were utilized to collect patient-reported information regarding health:

#### Knee injury and Osteoarthritis Outcome Score Joint Replacement (KOOS, JR.) [27]

The KOOS, JR. is a Likert-style questionnaire designed to evaluate patient's stiffness, pain (“twisting/pivoting”, “straightening”, “going up or down the stairs”, and “standing”), and functional ability (“rising from sitting” and “bending to the floor”). Patients indicate their level of stiffness and pain/difficulty performing these tasks based on the following options: “none”, “mild” “moderate”, “severe”, or “extreme”. The KOOS Jr. questions capture patient opinions up to 1 week prior to survey administration. Scores are transformed to a scale ranging from 0 to 100, with higher scores representing better knee function. The test has been validated against legacy measures, the Western Ontario and McMaster Universities Osteoarthritis Index (WOMAC) and full-length KOOS, which take considerably longer to administer [27]. This measure is also recommended for use with patients undergoing TKA in the perioperative period by the Centers for Medicare and Medicaid under the Comprehensive Care for Joint Replacement Model [28].

#### Patient-Reported Outcomes Measurement Information System (PROMIS) Global Health Instrument [29]

This instrument utilizes 10 items to provide an assessment of different components of patient quality-of-life. Items are used to provide raw scores for physical and mental health and raw response scores for the patient’s perception of overall health and social health. The physical health raw score (PROMIS PH) is calculated from items that ask patients to “rate” their physical health, fatigue, and pain while also considering the patient’s ability to carry out every day physical activities. Similarly, the mental health raw score (PROMIS MH) can be derived from items based on the patient’s general quality of life, mood and ability to think, social satisfaction, and susceptibility to emotional problems [30]. T-score tables are used for comparison of the physical and mental health raw scores to the general population [31]. After conversion, 50 is the mean, and converted t-scores that are 10 points below or above this number are understood to be 1 standard deviation away from the mean [30]. This allows for comparison of the mental and physical health scores to the general population with higher scores indicating better health. The social and overall health raw response scores provide insight into the patient’s perception at present, but these two items are not incorporated into composite scores [30]. For these two items, responses are recorded on a 5-point Likert Scale ranging from 1 = poor to 5 = excellent.

### Sample size

A sample size of 100 patients, achieves at least 80.9% power to detect an effect size (f^2^) of 0.10 attributable to 1 independent variable using an F-Test with a significance level (alpha) of 0.05. The variable tested can be adjusted for up to an additional 15 independent variable(s). An effect size of 0.15 can be detected with 93.4% power under similar assumptions. Cohen's f^2^ interpretation: 0.02 = small, 0.15 = medium, 0.35 = large. Thus, we were adequately powered to detect a small to medium effect size. Additional patients were recruited assuming that some patients would be lost to follow-up [32].

### Statistical analysis

Continuous variables are presented using the mean and standard deviation (SD) or median with 25th and 75th percentiles dependent on data distribution. Normality of continuous data was assessed using the Shapiro–Wilk test. Categorical variables are described using counts and percentages of non-missing data. Certain categorical variables were simplified into common subgroups to reduce categories and increase power. In order to measure internal consistency, Cronbach’s alpha coefficients were calculated for the primary patient-reported outcomes; values ≥ 0.70 generally indicate good reliability [33, 34].

Correlations among continuous preoperative variables were determined by calculating Pearson correlation coefficients to determine the linear relationship. Multivariable linear regression models were constructed for each 3-month postoperative primary outcome, including KOOS and PROMIS (GH and MH). The goal was to describe the independent association between each of resilience and pain catastrophizing and the outcome variable of interest. Based on our predetermined data collection procedures we anticipated the number of missing patients would be low. Therefore, we constructed three multivariable models for knee function, general physical health, and general mental health using only complete cases. Preoperative covariates were chosen for each of three models using univariable linear regression analyses to determine the relationship of *each* preoperative variable (described in Table 1) to each outcome. Each covariate with a significance level of *p* < 0.15 was considered and ultimately included in the multivariable models. Therefore, covariates for *each* model vary. This cutoff was selected to increase chances of including predictors in each final model that most appropriately explain each outcome in this cohort. Final models were assessed for multicollinearity using the variance inflation factor with a cutoff of 3. Assumptions for multiple regression were met for each outcome (i.e., normality of the residuals, homoscedasticity, and linearity). Model results are presented as the regression slope estimate with 95% confidence interval (CI).

**Table 1:** Population description (N = 117)

Variable	N (%)	Median (Q1, Q3)
Age, years		67.0 (59.0, 72.0)
Female sex	62 (53.0)	
White race	81 (70.1)	
Hispanic or Latino ethnicity^a^	1 (0.9)	
Private insurance	51 (43.6)	
Married or living as married	83 (70.9)	
Employed in or out of the home	46 (39.3)	
4-Year college or higher	67 (57.3)	
BMI, kg/m^2^		33.1 (29.0, 37.7)
ASA class		
1	2 (1.7)	
2	67 (57.3)	
3	48 (41.0)	
4–6	0 (0)	
Primary surgery	106 (90.6)	
Contralateral knee TKA	37 (31.6)	
Osteoarthritis etiology	113 (96.6)	
History of depression	36 (30.8)	
History of anxiety	22 (18.8)	
Smoking status		
Never smoker	74 (63.25)	
Former smoker	37 (31.62)	
Current smoker	6 (5.13)	
Pack years^b^		16.3 (6.0, 23.5)
History of diabetes	37 (31.6)	
History of hypertension	72 (61.5)	
History of cardiovascular disease	21 (17.9)	
History of back pain	68 (58.1)	
History of low back pain	56 (47.9)	
Pain rating		5.0 (3.0, 7.0)
BRS		4.0 (3.5, 4.3)
PCS		13 (5.0, 21.0)
KOOS IS		47.5 (39.6, 59.4)
PROMIS PH		39.8 (34.9, 44.9)
PROMIS MH		50.8 (45.8, 56.0)
PROMIS overall health rating		3.0 (3.0, 4.0)
PROMIS social activity rating		3.0 (2.0, 4.0)

Continuous variables are presented using the median (25th, 75th percentiles) and categorical data are displayed using counts with percentages for non-missing data unless otherwise noted

*BMI* body mass index, *ASA Class* American Society of Anesthesiologists physical status classification system, *BRS* Brief Resilience Score, *PCS* Pain Catastrophizing Scale, *Pain Rating* pain intensity on a 0–10-point scale, *KOOS IS* KOOS interval score, *PROMIS PH* PROMIS Global Physical Health T-score, *PROMIS MH* PROMIS Global Mental Health T-score, *PROMIS Overall Health Rating* raw response score from item 1 on PROMIS Global Health scale, *PROMIS Social Activity Rating* raw response score from remaining item 9 on PROMIS Global Health scale

^a^Hispanic or Latino Ethnicity was not included in further analyses due to low representation

^b^Pack years not available for every patient. N = 32

Pairwise differences within subject were calculated for baseline versus 3-month measurements for each outcome. The significance of each difference was tested using the Wilcoxon sign rank test for paired comparisons. A *p* < 0.05 was considered statistically significant unless otherwise indicated. Analyses were conducted using RStudio [35].

## Results

**Fig. 1:**
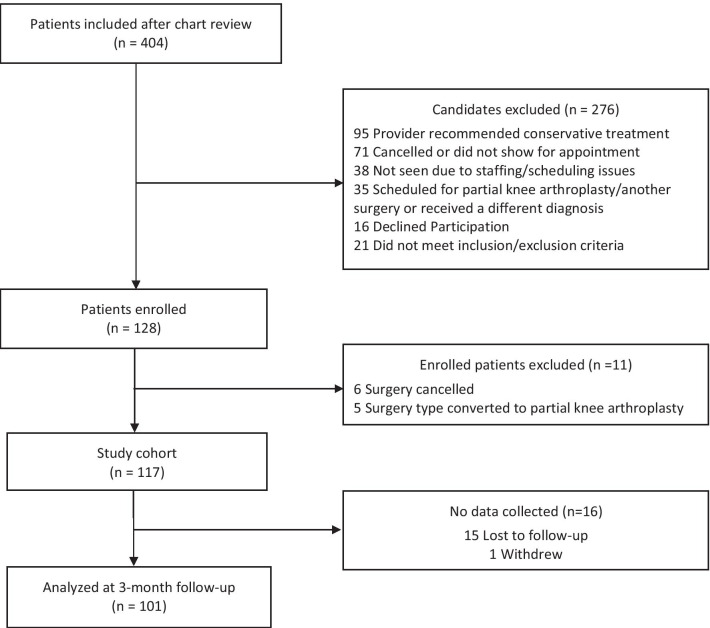
Flow diagram of patients in study

A total of 404 patients with upcoming clinic visits for surgery evaluation were screened in clinic based on chart review of eligibility criteria. Of those eligible patients, 276 candidates were excluded from inclusion or declined to participate based on rationale provided in Fig. 1. Of eligible subjects, 128 consented to participate; however, following consent, 11 patients were administratively withdrawn because of the change in their eligibility due to surgery (Fig. 1) leaving 117 patients in the final study cohort. The median number of days that baseline assessments were completed before surgery was 15 days (Q1–Q3: 12.0–22.0). At 3-months postoperative, the overall (clinic + phone/email/mail methods) response rate was 86.3%. Data were collected by phone/email/mail for 89.1% of the 101 patients analyzed at 3-month follow-up.

Overall, 53.0% of patients were women, and 70.1% of patients were white (Table 1). Of the 117 patients included in analyses, the median age was 67.0 years (Q1–Q3: 59.0–72.0). Additionally, 70.9% of patients were married and a history of depression was seen in 30.8% of patients while 18.8% of patients were found to have a history of anxiety. A history of diabetes and low back pain were reported in 31.6% and 47.9% of patients, respectively. Baseline measures of pain catastrophizing, resilience, pain, and general health are also included in Table 1 (Descriptive summaries of 3-month postoperative measures are included as Additional file 1: Table S1). The primary measures in the study demonstrated a satisfactory level of internal consistency with Cronbach’s alpha coefficients of 0.84 (BRS), 0.95 (PCS), 0.88 (KOOS), 0.71 (PROMIS PH), and 0.81 (PROMIS MH).

Correlations for baseline psychological variables with concurrent health and function measures are presented in Table 2. All correlations for pain catastrophizing and resilience across health and function measures at baseline were significant. Pain catastrophizing was negatively correlated with knee function, and general physical and mental health, while resilience was positively correlated with knee function and general physical and mental health prior to TKA.

**Table 2:** Correlation of baseline patient measures

	KOOS IS		PROMIS PH		PROMIS MH	
	r_p_	95% CI	r_p_	95% CI	r_p_	95% CI
PCS	− 0.63	− 0.51 to − 0.17	− 0.55	− 0.62 to − 0.32	− 0.46	− 0.52 to − 0.18
BRS	0.29	0.11 to 0.47	0.32	0.21 to 0.54	0.64	0.33 to 0.63

Values are given as the Pearson correlation coefficient with corresponding confidence interval

*BRS* Brief Resilience Score, *PCS* Pain Catastrophizing Scale, *KOOS IS* KOOS interval score, *PROMIS PH* PROMIS Global Physical Health T-score, *PROMIS MH* PROMIS Global Mental Health T- score

Simple linear regression and adjusted multiple variable regression models were used to determine whether baseline resilience was associated with knee function and general physical and mental health (Table 3). There was a significant association between preoperative resilience and knee function (β = 0.31, *p* = 0.002), general physical health (β = 0.40, *p* < 0.001), and mental health (β = 0.51, *p* < 0.001) in unadjusted models. After adjusting for pain catastrophizing and other covariates, the significant association of resilience persisted with 3-month knee function (β = 0.24, *p* = 0.019) and general physical health (β = 0.24, *p* = 0.013), but not with mental health at 3 months (β = 0.04, *p* = 0.738). The full multiple regression models are displayed in Additional file 2: Table S2, Additional file 3: Table S3, and Additional file 4: Table S4 as additional files.

**Table 3:** Regression models—unadjusted and adjusted associations between baseline resilience and function, physical health, and mental health at 3 months postoperative

Baseline resilience	Unadjusted		Adjusted for baseline pain catastrophizing		Adjusted for baseline pain catastrophizing and covariates	
	β (95% CI)	*p* value	β (95% CI)	*p* value	β (95% CI)	*p* value
3-Month knee function	0.31 (0.11–0.50)	0.002	− 0.19 (− 0.02 to 0.40)	0.074	0.24^a^ (0.04–0.44)	0.019
3-Month physical health	0.40 (0.21–0.58)	< 0.001	0.23 (0.04–0.42)	0.018	0.24^b^ (0.05–0.42)	0.013
3-Month mental health	0.51 (0.33–0.68)	< 0.001	0.43 (0.24–0.62)	< 0.001	0.04^c^ (− 0.18 to 0.25)	0.738

Models adjusted for preoperative covariates based on *p* < 0.15 in univariable analyses:

^a^Baseline KOOS IS, pain rating, race, education level, employment status, BMI, and arthritis etiology. Adjusted R-squared = 0.25

^b^Baseline PROMIS PH, pain rating, age, race, education level, employment status, procedure type, BMI, history of contralateral TKA, diabetes, hypertension, and low back pain. Adjusted R-squared = 0.40

^c^Baseline PROMIS MH, pain rating, age, education level, primary insurance, procedure type, history of depression, anxiety, cardiovascular disease, and of low back pain. Adjusted R-squared = 0.49

## Discussion

In the current study, we investigated the association between preoperative resilience and postoperative knee function and general health among patients undergoing TKA. The association has not been widely explored in this patient population and to-date has not accounted for a known negative psychological construct, pain catastrophizing. We found preoperative resilience was positively correlated with preoperative knee function and general health. Importantly, our findings suggest that baseline resilience was predictive of 3-month knee function and general physical health and the significant association persisted after adjusting for pain catastrophizing and other patient covariates. This positive psychological construct may be an important area of focus for future intervention in support of optimizing patient outcomes after TKA.

Few studies of patients with TKA have explored resilience and postoperative outcomes and comparisons are limited due to differences in populations studied. Our findings specific to patients with TKA demonstrated a statistically significant association between resilience and physical function; clinical significance was marginal as in previous studies [27, 36]. Rebagliati et al. [18] studied patients who underwent elective *or* traumatic hip and knee surgery. Though the study found no relationship between resilience (measured by the Resilience Scale [37]) and functional independence, the number of patients with knee surgery in this cohort was unspecified which limits and our ability to compare findings. A second study by this research group using the same resilience scale found that the level of presurgical resilience did not relate to functional independence for patients who had undergone elective joint replacement surgery [19]. This study of 80 patients who underwent joint replacement due to *fracture* concluded that patients who were less resilient when measured preoperatively were less likely to be functionally independent post-operatively; however only seven of the 80 who participated in the study had TKA and other baseline characteristics were not reported for patients with TKA versus THA. In a recent study, Magaldi et al. [20] demonstrated that baseline resilience (measured by the BRS) was not associated with knee function measured by KOOS JR. but was associated with PROMIS physical health and mental health scores at 3-month and 12-month follow-up. We hypothesize that our 3-month findings differ because we adjusted for pain catastrophizing in the current study which has a solid foundation of evidence for its influence on outcomes after TKA.

Based on our findings, we also postulate that patients with positive perceptions of their recovery process are able to handle the mental and physical stressors of surgery better than those with negative perceptions. Their opinions of recovery are likely based on previous adverse experiences, daily recovery progress, as well as traits that cumulatively guide the patient toward specific emotional and behavioral responses [17, 38]. Some patients may embody a trait known as “committed action”, which prompts them to *intentionally* pursue physically and mentally challenging tasks [39]. A patient who believes they are resilient will likely have higher levels of self-efficacy and will be more determined to complete what they perceive to be difficult physical tasks following surgery [40]. Alternatively, these resilient patients may not view surgery as an insurmountable obstacle, believing that the process of recovery is simply part of a necessary routine [6]. Furthermore, resilient patients may have a more positive evaluation of their perceived function during the recovery process because they self-identify as resilient patients, necessitating an equivalent self-evaluation to prevent cognitive dissonance. Finally, these resilient patients may also have social support networks that positively influence their progress perioperatively [38].

The relationship between pain catastrophizing and postoperative knee function, specifically in persons with TKA has received much attention over the last 2 decades. Three studies have found significant relationships between pain catastrophizing and function as measured by the WOMAC knee function subscale and postoperative function during follow-up as early as 6-weeks [5], 6-months [41], and 1-year [42] after adjustment. A recent study found that patients with higher PCS scores (> 21) showed more improvement than patients with lower PCS scores (< 11) with regard to function on the Oxford Knee Score at 12-months postoperative [43]. In the current study, pain catastrophizing was negatively correlated with knee function and the PROMIS general health assessments. This is the first study we are aware of to analyze pain catastrophizing and associations with PROMIS physical health and mental health components. We used this as an important signal of influence on recovery and why pain catastrophizing should be adjusted for in cohort studies examining outcomes of patients after TKA.

Considering the evidence to-date regarding psychological health and the relationship with outcomes after TKA, we suggest that further research is warranted in this field to elicit other key factors influencing functional recovery [44–47]. Determining which factors significantly and *consistently* predict postoperative outcomes will allow clinicians to gain insights about their patients’ potential functional trajectory that may not have been considered previously. As suggested in recent literature, the broader understanding of the patient experience beyond negative psychosocial factors may hold the key to eliciting modifiable risk factors for patients undergoing TKA [39]. The current study is powerful because it highlights a rarely assessed concept that has potential for integration into future multimodal predictions tools for outcomes related to TKA [39]. In addition to defining the mechanisms behind the relationships between resilience and function as presented above, additional research in this area can also expand on the role of new psychosocial variables in the context of patient satisfaction in an increasingly patient-centered and patient-evaluated health care system. In addition to the development of new preoperative tools, future directions in the field may target perioperative *interventions* that address key psychological constructs. Two recent studies sought to improve functional outcomes in pain catastrophizing patients undergoing TKA using pain coping skills training [48] and cognitive behavioral therapy [49]; however, neither study was able to demonstrate that the selected interventions were superior to usual care. To our knowledge, there are no studies in the arthroplasty literature that have tested interventions for patients with low resilience in an effort to improve postoperative function, general health or quality of life.

The current study is limited mostly by factors which were related to the most efficient and practical methods of collecting data from a prospective patient cohort in this academic medical center [50, 51]. Patients were excluded based on English written or verbal proficiency. Standardized measures were not available in the various languages present in this geography and interpreters were not available to study staff for each data collection time point, necessitating this limitation to generalizability. Neither the prospective data collection or electronic medical record included duration of the disease or stage of the disease (e.g., the Kellgren Lawrence classification scores), limiting our ability to examine any differences in outcomes by these variables. Additionally, although patients were generally managed with pain medications under similar drug classes, the postoperative regimens for individual patients may have varied outside of the hospital setting. This study also has strengths that should be considered. Enrollment was conducted primarily by the same individual, which contributes to the consistency of delivering study details, answering patient questions and obtaining consent. All patients provided complete contact information (email address, phone number, and mailing addresses) upon enrolling in the study which facilitated subsequent data collection. Patients in the study were screened consecutively to limit selection bias. Additionally, the patients of four providers within the practice were utilized in order to increase generalization of the results. To prevent loss of patients to follow-up, the team prioritized the patients’ post-surgery visits in clinic to complete follow-up measures despite that this may have introduced social desirability bias. Investigators made an effort to limit unmeasured confounding by considering other variables available in the medical record that may influence associations.

## Conclusions

In conclusion, our study contributes new information on preoperative resilience and its association with 3-month patient-reported outcomes of knee function and physical health. To our knowledge, this is the first study of patients with TKA that suggests this association persists after accounting for pain catastrophizing, sociodemographic, and clinical factors. The results of this study reinforce the importance of assessing preoperative psychological variables prior to TKA. Additional research is needed to support the development and implementation of resilience-related interventions in patients undergoing TKA to evaluate the potential for improvement in knee function and physical health following surgery.

## Supplementary Information

**Additional file 1.** **Supplemental Table 1.** Patient-Reported Outcome Measure Scores at 3-Months Postoperative and Change from Baseline in Study Population.

**Additional file 2.** **Supplemental Table 2.** Unadjusted and Adjusted Models for KOOS IS at 3-Months Postoperative.

**Additional file 3.** **Supplemental Table 3.** Unadjusted and Adjusted Models for PROMIS PH at 3-Months Postoperative.

**Additional file 4.** **Supplemental Table 4.** Unadjusted and Adjusted Models for PROMIS MH at 3-Months Postoperative.

## Data Availability

The datasets used and/or analyzed during the current study are available from the corresponding author on reasonable request.

## Abbreviations

ASA: American Society of Anesthesiologists
B: Unstandardized beta
BMI: Body mass index
BRS: Brief Resilience Score
CI: Confidence interval
IRB: Institutional Review Board
IQR: Interquartile range
KOOS IS: KOOS Interval Score
KOOS, Jr.: Knee injury and Osteoarthritis Outcome Score Joint Replacement
NRS: Pain Numeric Rating Scale
PCS: Pain Catastrophizing Scale
PROMIS: Patient-Reported Outcomes Measurement Information System
PROMIS MH: PROMIS Global Mental Health T-score
PROMIS PH: PROMIS Global Physical Health T-score
Q1: 25Th Quartile
Q3: 75Th Quartile
r_p_: Pearson correlation coefficient
SD: Standard deviation
SF: Short Form
TKA: Total knee arthroplasty
WOMAC: Western Ontario and McMaster Universities Osteoarthritis Index
β: Standardized beta

## References

[1] Sloan M, Premkumar A, Sheth N (2018). Projected volume of primary total joint arthroplasty in the U.S., 2014 to 2030. J Bone Joint Surg Am.

[2] Van Manen MD, Nace J, Mont MA (2012). Management of primary knee osteoarthritis and indications for total knee arthroplasty for general practitioners. J Am Osteopath Assoc.

[3] Mahomed NN, Barrett J, Katz JN, Baron JA, Wright J, Losina E (2005). Epidemiology of total knee replacement in the United States Medicare population. J Bone Joint Surg Am.

[4] Jevsevar DS (2013). Treatment of osteoarthritis of the knee: evidence-based guideline, 2nd edition. J Am Acad Orthop Surg.

[5] Sullivan M, Tanzer M, Stanish W, Fallaha M, Keefe FJ, Simmonds M, Dunbar M (2009). Psychological determinants of problematic outcomes following total knee arthroplasty. Pain.

[6] Sturgeon JA, Zautra AJ (2013). Psychological resilience, pain catastrophizing, and positive emotions: perspectives on comprehensive modeling of individual pain adaptation. Curr Pain Headache Rep.

[7] Burns LC, Ritvo SE, Ferguson MK, Clarke H, Seltzer Z, Katz J (2015). Pain catastrophizing as a risk factor for chronic pain after total knee arthroplasty: a systematic review. J Pain Res.

[8] Lungu E, Vendittoli PA, Desmeules F (2016). Preoperative determinants of patient-reported pain and physical function levels following total knee arthroplasty: a systematic review. Open Orthop J.

[9] Edwards RR, Haythornthwaite JA, Smith MT, Klick B, Katz JN (2009). Catastrophizing and depressive symptoms as prospective predictors of outcomes following total knee replacement. Pain Res Manag.

[10] Sturgeon JA, Zautra AJ (2010). Resilience: a new paradigm for adaptation to chronic pain. Curr Pain Headache Rep.

[11] Smith BW, Dalen J, Wiggins K, Tooley E, Christopher P, Bernard J (2008). The brief resilience scale: assessing the ability to bounce back. Int J Behav Med.

[12] Zautra AJ, Arewasikporn A, Davis MC (2010). Resilience: promoting well-being through recovery, sustainability, and growth. Res Hum Dev.

[13] Suffeda A, Meissner W, Rosendahl J, Guntinas-Lichius O (2016). Influence of depression, catastrophizing, anxiety, and resilience on postoperative pain at the first day after otolaryngological surgery: a prospective single center cohort observational study. Hanaoka. K, ed. Medicine.

[14] Duggal D, Sacks-Zimmerman A, Liberta T (2016). The impact of hope and resilience on multiple factors in neurosurgical patients. Muacevic A, Adler JR, eds. Cureus.

[15] Wu HC (2011). The protective effects of resilience and hope on quality of life of the families coping with the criminal traumatisation of one of its members. J Clin Nurs.

[16] Heathcote K, Wullschleger M, Sun J (2019). The effectiveness of multi-dimensional resilience rehabilitation programs after traumatic physical injuries: a systematic review and meta-analysis. Disabil Rehabil.

[17] Ong AD, Zautra AJ, Reid MC (2010). Psychological resilience predicts decreases in pain catastrophizing through positive emotions. Psychol Aging.

[18] Rebagliati GA, Sciume L, Iannello P, Mottini A, Antonietti A, Caserta VA, Gattoronchieri V, Panella L, Callegari C (2016). Frailty and resilience in an older population. The role of resilience during rehabilitation after orthopedic surgery in geriatric patients with multiple comorbidities. Funct Neurol.

[19] Sciume L, Rebagliati G, Iannello P, Mottini A, Alessandro A, Caserta AV, Gattoronchieri V, Panella L (2018). Rehabilitation after urgent or elective orthopedic surgery: the role of resilience in elderly patients. Rehabil Nurs.

[20] Magaldi RJ, Staff I, Stovall AE, Stohler SA, Lewis CG (2019). Impact of resilience on outcomes of total knee arthroplasty. J Arthroplasty.

[21] Harris PA, Taylor R, Thielke R, Payne J, Gonzalez N, Conde JG (2009). Research electronic data capture (REDCap)—a metadata-driven methodology and workflow process for providing translational research informatics support. J Biomed Inform.

[22] Schreurs BW, Hannink G (2017). Total joint arthroplasty in younger patients: heading for trouble?. The Lancet.

[23] Haefeli M, Elfering A (2006). Pain assessment. Eur Spine J.

[24] What is Cardiovascular Disease? American Heart Association. Updated May 31, 2017. https://www.heart.org/en/health-topics/consumer-healthcare/what-is-cardiovascular-disease. Accessed 15 Nov 2019.

[25] Hurwitz EE, Simon M, Vinta SR, Zehm CF, Shabot SM, Minhajuddin A, Abouleish AE (2017). Adding examples to the ASA-physical status classification improves correct assignment to patients. Anesthesiology.

[26] Sullivan MJL, Bishop SR, Pivik J (1995). The pain catastrophizing scale: development and validation. Psychol Assess.

[27] Lyman S, Lee Y-Y, Franklin PD, Li W, Cross MB, Padgett DE (2016). Validation of the KOOS, JR: a short-form knee arthroplasty outcomes survey. Clin Orthop Relat Res.

[28] Centers for Medicare & Medicaid Services (CMS), HHS (2015). Medicare program; comprehensive care for joint replacement payment model for acute care hospitals furnishing lower extremity joint replacement services. Final rule. Fed Regist.

[29] Cella D, Riley W, Stone A, Rothrock N, Reeve B, Yount S, Amtmann D, Bode R, Buysse D, Choi S, Cook K, Devellis R, DeWalt D, Fries JF, Gershon R, Hahn EA, Lai J-S, Pilkonis P, Revicki D, Rose M, Weinfurt K, Hays R, PROMIS Cooperative Group (2010). The Patient-Reported Outcomes Measurement Information System (PROMIS) developed and tested its first wave of adult self-reported health outcome item banks: 2005–2008. J Clin Epidemiol.

[30] Global Health: a brief guide to the PROMIS© Global Health instruments. Health Measures. March 6, 2017. http://www.healthmeasures.net/index.php?option=com_instruments&view=measure&id=778&Itemid=992. Accessed 20 Jan 2019.

[31] Hays RD, Bjorner JB, Revicki DA, Spritzer KL, Cella D (2009). Development of physical and mental health summary scores from the patient-reported outcomes measurement information system (PROMIS) global items. Qual Life Res.

[32] Cohen J (1988). Statistical power analysis for the behavioral sciences.

[33] Cronbach LJ (1951). Coefficient alpha and the internal structure of tests. Psychometrika.

[34] Kline P (1999). The handbook of psychological testing.

[35] RStudio Team (2016). RStudio: integrated development for R.

[36] Khalil LS, Darrith B, Franovic S, Davis JJ, Weir RM, Banka TR (2020). Patient-Reported Outcomes Measurement Information System (PROMIS) global health short forms demonstrate responsiveness in patients undergoing knee arthroplasty. J Arthroplasty.

[37] Wagnild GM, Young HM (1993). Development and psychometric evaluation of the resilience scale. J Nurs Meas.

[38] Sturgeon JA, Zautra AJ (2016). Social pain and physical pain: shared paths to resilience. Pain Manag.

[39] Ditton E, Johnson S, Hodyl N, Flynn T, Pollack M, Ribbons K, Walker FR, Nilsson M (2020). Improving patient outcomes following total knee arthroplasty: identifying rehabilitation pathways based on modifiable psychological risk and resilience factors. Front Psychol.

[40] Heathcote K, Wullschleger M, Sun J (2019). The effectiveness of multi-dimensional resilience rehabilitation programs after traumatic physical injuries: a systematic review and meta-analysis. Disabil Rehabil.

[41] Riddle DL, Wade JB, Jiranek WA, Kong X (2010). Preoperative pain catastrophizing predicts pain outcome after knee arthroplasty. Clin Orthop Relat Res.

[42] Sullivan M, Tanzer M, Reardon G, Amirault D, Dunbar M, Stanish W (2011). The role of presurgical expectancies in predicting pain and function one year following total knee arthroplasty. Pain.

[43] Birch S, Stilling M, Mechlenburg I, Hansen TB (2019). The association between pain catastrophizing, physical function and pain in a cohort of patients undergoing knee arthroplasty. BMC Musculoskelet Disord.

[44] Ali A, Lindstrand A, Sundberg M, Flivik G (2017). Preoperative anxiety and depression correlate with dissatisfaction after total knee arthroplasty: a prospective longitudinal cohort study of 186 patients, with 4-year follow-up. J Arthroplasty.

[45] Khatib Y, Madan A, Naylor JM, Harris IA (2015). Do psychological factors predict poor outcome in patients undergoing TKA? A systematic review. Clin Orthop Relat Res.

[46] Clement N (2013). Patient factors that influence the outcome of total knee replacement: a critical review of the literature. OA Orthop.

[47] Vissers MM, Bussmann JB, Verhaar JAN, Busschbach JJV, Bierma-Zeinstra SMA, Reijman M (2012). Psychological factors affecting the outcome of total hip and knee arthroplasty: a systematic review. Semin Arthritis Rheum.

[48] Riddle DL, Keefe FJ, Ang DC, Slover J, Jensen MP, Bair MJ, Kroenke K, Perera RA, Reed SD, McKee D, Dumenci L (2019). Pain coping skills training for patients who catastrophize about pain prior to knee arthroplasty: a multisite randomized clinical trial. J Bone Joint Surg Am.

[49] Birch S, Stilling M, Mechlenburg I, Hansen TB (2020). No effect of cognitive behavioral patient education for patients with pain catastrophizing before total knee arthroplasty: a randomized controlled trial. Acta Orthop.

[50] Althubaiti A (2016). Information bias in health research: definition, pitfalls, and adjustment methods. J Multidiscip Healthc.

[51] Ranstam J (2008). Bias in observational studies. Acta Radiol.

