# Supplementary material for: Resilience and pain catastrophizing among patients with total knee arthroplasty: a cohort study to examine psychological constructs as predictors of post-operative outcomes
Source: Health Qual Life Outcomes. 2021 May 1;19:136. doi: 10.1186/s12955-021-01772-2 (PMC8088639; doi:10.1186/s12955-021-01772-2)
Supplement: Supplementary file 1 — Additional file 1. Supplemental Table 1. Patient-Reported Outcome Measure Scores at 3-Months Postoperative and Change from Baseline in Study Population. [file 12955_2021_1772_MOESM1_ESM.docx]

| **Supplemental Table 1.** Patient-Reported Outcome Measure Scores at 3-Months Postoperative and Change from Baseline in Study Population. | | | | |
| --- | --- | --- | --- | --- |
|  | 3-Months Postoperative | ∆ from Baseline | |  |
| **Variable** | Median (Q1, Q3) | Median (Q1, Q3) | | P-value* |
| Pain Rating | 1.0 (0, 3.0) | | -2.0 (-4.0, 0) | <0.001 |
| BRS | 4.0 (3.6, 4.7) | | 0 (-0.2, 0.08) | 0.167 |
| PCS | 1.0 (0, 7.0) | | -5 (-13.0, 0) | <0.001 |
| KOOS IS | 66.0 (59.4, 76.3) | | 22.5 (10.7, 31.1) | <0.001 |
| PROMIS PH | 47.7 (42.3, 54.1) | | 7.5 (2.8, 11.1) | <0.001 |
| PROMIS MH | 50.8 (45.8, 59.0) | | 2.3 (-2.5, 5.20) | 0.021 |
| *BRS = Brief Resilience Score, PCS = Pain Catastrophizing Scale, Pain Rating = Pain intensity on 0–10-point scale, KOOS IS = KOOS interval score, PROMIS PH = PROMIS Global Physical Health T-score, PROMIS MH = PROMIS Global Mental Health T- score,* ∆ = Change.  *Baseline versus 3-month measurements (Wilcoxon Signed Rank test for paired comparisons). | | | | |
