# Supplementary material for: Resilience and pain catastrophizing among patients with total knee arthroplasty: a cohort study to examine psychological constructs as predictors of post-operative outcomes
Source: Health Qual Life Outcomes. 2021 May 1;19:136. doi: 10.1186/s12955-021-01772-2 (PMC8088639; doi:10.1186/s12955-021-01772-2)
Supplement: Supplementary file 2 — Additional file 2. Supplemental Table 2. Unadjusted and Adjusted Models for KOOS IS at 3-Months Postoperative. [file 12955_2021_1772_MOESM2_ESM.docx]

| **Supplemental Table 2. Unadjusted and Adjusted Models for KOOS IS at 3-Months Postoperative.** | | | | |
| --- | --- | --- | --- | --- |
|  | | | | |
|  | Unadjusted | | Adjusted | |
| Baseline | ***β (95% CI)** | **P-value** | ***β (95% CI)** | **P-value** |
| KOOS IS Baseline | 0.39 (0.20 to 0.58) | < 0.001 | -0.04 (-0.32 to 0.24) | 0.773 |
| PCS | -0.36 (-0.55 to -0.17) | < 0.001 | -0.08 (-0.32 to 0.17) | 0.543 |
| **BRS** | **0.31 (0.11 to 0.50)** | **0.002** | **0.24 (0.04 to 0.44)** | **0.019** |
| **Pain Rating** | **-0.43 (-0.61 to -0.24)** | **< 0.001** | **-0.33 ( -0.57 to -0.08)** | **0.009** |
| Age, years | 0.05 (-0.17 to 0.24) | 0.727 |  | |
| Female) vs Male *(ref.)* | 0.79 (-4.63 to 6.20) | 0.773 |  | |
| Nonwhite vs White  (ref.) | -4.38 (-10.18 to 1.43) | 0.138 | 0.03 (-0.37 to 0.43) | 0.887 |
| Unmarried vs Married  (*ref*.) | 0.35 ( -5.76 to 6.47) | 0.909 |  | |
| Associate’s Degree or  below vs College  education (*ref*.) | -6.14 (-11.52 to -0.77) | 0.026 | -0.14 (-0.53 to 0.26) | 0.484 |
| Unemployed vs  Employed (*ref*.) | 5.33 (-0.06 to 10.72) | 0.052 | 0.24 (-0.12 to 0.59) | 0.191 |
| Government vs Private  (*ref*.) Insurance | 1.70 ( -3.74 to 7.15) | 0.536 |  | |
| Revision vs Primary  (*ref*.) | 0.01 (-9.05 to 9.07) | 0.998 |  | |
| Contralateral TKA | 4.00 (-1.82 to 9.81) | 0.176 |  | |
| BMI, kg/m2 | -0.18 ( -0.38 to 0.02) | 0.071 | -0.16 (-0.35 to 0.02) | 0.085 |
| ASA 3 vs ASA 1 or 2 (*ref*.) | -1.26 ( -6.76 to 4.25) | 0.652 |  | |
| **Osteoarthritis** | **14.05 (0.46 to 27.65)** | **0.043** | **0.98 (0.03 to 1.92)** | **0.043** |
| Depression | 0.28 (-5.84 to 6.40) | 0.928 |  | |
| Anxiety | 3.92 (-3.45 to 11.29) | 0.294 |  | |
| Diabetes | -4.03 (-9.66 to 1.61) | 0.159 |  | |
| Hypertension | -1.57 (-7.10 to 3.95) | 0.574 |  | |
| Cardiovascular Disease | -3.61 (-10.37 to 3.14) | 0.291 |  | |
| Low Back Pain | -1.34 (-6.76 to 4.08) | 0.626 |  | |
| Smoking Status: Never  smoker (*ref*.) | 3.50 (-2.10 to 9.11) | 0.218 |  | |
| Note:  Model adjusted for baseline KOOS IS, pain rating, race, education level, employment status, BMI, and arthritis  etiology. Adjusted R-squared =0.25.  *Abbreviations: BRS = Brief Resilience Score, PCS = Pain Catastrophizing Scale, KOOS IS = KOOS interval score, ref=reference variable, CI=Confidence Interval.*  * Standardized regression coefficients are presented for continuous variables. Coefficients for categorical variables  remain unstandardized. | | | | |
