# Supplementary material for: Resilience and pain catastrophizing among patients with total knee arthroplasty: a cohort study to examine psychological constructs as predictors of post-operative outcomes
Source: Health Qual Life Outcomes. 2021 May 1;19:136. doi: 10.1186/s12955-021-01772-2 (PMC8088639; doi:10.1186/s12955-021-01772-2)
Supplement: Supplementary file 3 — Additional file 3. Supplemental Table 3. Unadjusted and Adjusted Models for PROMIS PH at 3-Months Postoperative. [file 12955_2021_1772_MOESM3_ESM.docx]

| **Supplemental Table 3. Unadjusted and Adjusted Models for PROMIS PH at 3-Months Postoperative.** | | | | |
| --- | --- | --- | --- | --- |
|  | | | | |
|  | Unadjusted | | Adjusted | |
| Baseline | ***β (95% CI)** | **P-value** | ***β (95% CI)** | **P-value** |
| **PROMIS PH Baseline** | **0.60 (0.43 to 0.77)** | **< 0.001** | **0.26 (0.02 to 0.50)** | **0.031** |
| PCS | -0.49 (-0.67 to -0.32) | < 0.001 | -0.11 (-0.33 to 0.11) | 0.313 |
| **BRS** | **0.40 (0.21 to 0.58)** | **< 0.001** | **0.24 (0.05 to 0.42)** | **0.013** |
| Pain rating | -0.45 (-0.63 to -0.27) | < 0.001 | -0.21 (-0.45 to 0.03) | 0.088 |
| Age, years | 0.27 (0.07 to 0.46) | 0.008 | -0.03 (-0.24 to 0.18) | 0.764 |
| Female) vs Male *(ref.)* | -0.71 (-4.05 to 2.63) | 0.674 |  | |
| Nonwhite vs White  (ref.) | -3.39 (-6.94 to 0.17) | 0.062 | -0.07 (-0.45 to 0.31) | 0.711 |
| Unmarried vs Married  (*ref*.) | -1.11 ( -4.88 to 2.65) | 0.558 |  | |
| Associate’s Degree or  below vs College  education (*ref*.) | -2.91 (-6.26 to 0.44) | 0.088 | 0.13 (-0.22 to 0.49) | 0.460 |
| Unemployed vs  Employed (*ref*.) | 2.88 (-0.46 to 6.22) | 0.090 | 0.30 (-0.05 to 0.65) | 0.093 |
| Government vs Private  (*ref*.) Insurance | 2.39 (-0.94 to 5.73) | 0.158 |  | |
| Revision vs Primary  (*ref*.) | -4.57 (-10.09 to 0.94) | 0.103 | -0.04 (-0.61 to 0.53) | 0.891 |
| Contralateral TKA | 2.69 (-0.89 to 6.27) | 0.139 | 8.5e-4 (-0.41 to 0.41) | 0.997 |
| BMI, kg/m2 | -0.30 (-0.49 to -0.11) | 0.002 | -0.12 (-0.30 to 0.07) | 0.208 |
| ASA 3 vs ASA 1 or 2 (*ref*.) | -2.09 (-5.46 to 1.29) | 0.223 |  | |
| Osteoarthritis | 6.20 (-2.28 to 14.67) | 0.150 |  | |
| Depression | -1.86 (-5.62 to 1.89) | 0.327 |  | |
| Anxiety | 1.19 (-3.38 to 5.75) | 0.608 |  | |
| Diabetes | -3.62 (-7.05 to -0.18) | 0.039 | -0.15 (-0.53 to 0.23) | 0.438 |
| Hypertension | -3.98 (-7.30 to -0.66) | 0.019 | -0.22 (-0.58 to 0.14) | 0.231 |
| Cardiovascular Disease | -1.88 (-6.05 to 2.29) | 0.374 |  | |
| Low Back Pain | -4.40 (-7.63 to -1.17) | 0.008 | -0.10 (-0.46 to 0.26) | 0.581 |
| Smoking Status: Never  smoker (*ref*.) | 0.798 (-3.04 to 3.94) | 0.798 |  | |
| Model adjusted for baseline PROMIS PH, pain rating, age, race, education level, employment status, procedure type, BMI, history of contralateral TKA, diabetes, hypertension, and low back pain. Adjusted R-squared =0.40.  *Abbreviations: BRS = Brief Resilience Score, PCS = Pain Catastrophizing Scale, KOOS IS = KOOS interval score, ref=reference variable, CI=Confidence Interval.*  * Standardized regression coefficients are presented for continuous variables. Coefficients for categorical variables  remain unstandardized. | | | | |
