# Supplementary material for: Resilience and pain catastrophizing among patients with total knee arthroplasty: a cohort study to examine psychological constructs as predictors of post-operative outcomes
Source: Health Qual Life Outcomes. 2021 May 1;19:136. doi: 10.1186/s12955-021-01772-2 (PMC8088639; doi:10.1186/s12955-021-01772-2)
Supplement: Supplementary file 4 — Additional file 4. Supplemental Table 4. Unadjusted and Adjusted Models for PROMIS MH at 3-Months Postoperative. [file 12955_2021_1772_MOESM4_ESM.docx]

| **Supplemental Table 4. Unadjusted and Adjusted Models for PROMIS MH at 3-Months Postoperative.** | | | | |
| --- | --- | --- | --- | --- |
|  | | | | |
|  | Unadjusted | | Adjusted | |
| Baseline | ***β (95% CI)** | **P-value** | ***β (95% CI)** | **P-value** |
| **PROMIS MH Baseline** | **0.68 (0.55 to 0.82)** | **< 0.001** | **0.57 (0.37 to 0.76)** | **<0.001** |
| PCS | -0.37 (-0.56 to -0.18) | < 0.001 | 0.01 (-0.19 to -0.21) | 0.919 |
| BRS | 0.51 (0.33 to 0.68) | < 0.001 | 0.04 (-0.18 to 0.25) | 0.738 |
| Pain rating | -0.29 (-0.48 to -0.09) | 0.004 | -0.08 (-0.26 to 0.10) | 0.360 |
| Age, years | 0.19 (-0.01 to 0.39) | 0.062 | 0.02 (-0.16 to 0.20) | 0.832 |
| Female) vs Male *(ref.)* | -0.54 (-4.32 to 3.25) | 0.780 |  | |
| Nonwhite vs White  (ref.) | -1.31 (-5.41 to 2.79) | 0.528 |  | |
| Unmarried vs Married  (*ref*.) | -2.16 ( -6.42 to 2.10) | 0.317 |  | |
| Associate’s Degree or  below vs College  education (*ref*.) | -3.32 (-7.12 to 0.48) | 0.086 | -0.14 (-0.46 to 0.17) | 0.370 |
| Unemployed vs  Employed (*ref*.) | 2.69 (-1.11 to 6.50) | 0.164 |  | |
| Government vs Private  (*ref*.) Insurance | 5.03 (1.35 to 8.72) | 0.008 | 0.05 (-0.28 to 0.38) | 0.772 |
| Revision vs Primary  (*ref*.) | -5.82 (-12.06 to 0.41) | 0.067 | -0.34 ( -0.83 to 0.14) | 0.162 |
| Contralateral TKA | 2.91(-1.16 to 6.97) | 0.159 |  | |
| BMI, kg/m2 | -0.09 ( -0.28 to 0.11) | 0.392 |  | |
| ASA 3 vs ASA 1 or 2 (*ref*.) | -2.22 ( -6.05 to 1.62) | 0.254 |  | |
| Osteoarthritis | 4.18 (-5.49 to 13.86) | 0.393 |  | |
| Depression | -6.42 (-10.50 to -2.33) | 0.002 | -0.15 (-0.54 to 0.23) | 0.435 |
| Anxiety | -5.79 (-10.85 to -0.73) | 0.025 | -0.30 (-0.72 to 0.12) | 0.158 |
| Diabetes | -2.56 (-6.50 to 1.39) | 0.202 |  | |
| Hypertension | -2.65 (-6.49 to 1.18) | 0.173 |  | |
| Cardiovascular Disease | -3.66 (-8.35 to 1.04) | 0.126 | -0.19 (-0.57 to 0.19) | 0.312 |
| Low Back Pain | -4.48 (-8.17 to -0.79) | 0.018 | -0.07 (-0.37 to 0.24) | 0.667 |
| Smoking Status: Never  smoker (*ref*.) | -1.40 ( -5.34 to 2.55) | 0.484 |  | |
| Model adjusted for baseline PROMIS MH, pain rating, age, education level, primary insurance, procedure type, history of depression, anxiety, cardiovascular disease, and of low back pain. Adjusted R-squared = 0.49  *Abbreviations: BRS = Brief Resilience Score, PCS = Pain Catastrophizing Scale, KOOS IS = KOOS interval score, ref=reference variable, CI=Confidence Interval.*  * Standardized regression coefficients are presented for continuous variables. Coefficients for categorical variables  remain unstandardized. | | | | |
